# Caffeic Acid but Not Cinnamic Acid Mitigates Impairing Effects of Indomethacin in Porcine Stomach Epithelium In Vitro

**DOI:** 10.3390/biom16101512

**Published:** 2026-10-18

**Authors:** Marie-Luise Vollstädt, Laura Stein, Elisa Boehm, Linda Droessler, Salah Amasheh

**Affiliations:** Institute of Veterinary Physiology, School of Veterinary Medicine, Freie Universität Berlin, 14163 Berlin, Germany

**Keywords:** tight junction, stomach, cinnamic acid, caffeic acid, Ussing chamber

## Abstract

In recent years, a growing interest in plant-based medicine has been observed, mainly due to a wide variety of secondary plant compounds and their health-promoting properties. Multiple health-supporting qualities have been described, but much remains to be discovered. In this study, two of the most abundant polyphenols, cinnamic acid and caffeic acid, were analyzed regarding their effects on the stomach mucosal epithelium ex vivo. To accomplish this, Ussing chamber experiments with native porcine stomach epithelial tissue specimens were performed, recording transepithelial electrical resistance, followed by immunoblotting and confocal laser scanning immunofluorescence microscopy of tight junction proteins. Additionally, to analyze potential effects not only on the physiological mucosa but also on challenged stomach epithelium, an indomethacin-induced gastric epithelial barrier impairment model for Ussing chamber experiments was employed. In this study, no effects of cinnamic acid were observed, whereas caffeic acid mitigated the impairing effects of indomethacin in porcine stomach epithelial tissue regarding changes in transepithelial resistance as well as the composition of tight junction proteins. These results indicate a strengthening effect of caffeic acid on gastric mucosa and imply benefits of caffeic acid as a plant-based drug in the prevention and treatment of gastritis.

## 1. Introduction

The stomach is lined with a columnar epithelial cell monolayer, which is apicolaterally connected by tight junctions (TJs), controlling the paracellular permeability. A key role as a structural correlate of paracellular permeability has been attributed to TJ proteins, primarily represented by the family of claudins, which are tetraspan transmembrane proteins with C-terminal and N-terminal cytosolic domains, one intracellular loop, and two extracellular loops [1]. A binding domain within the C-terminal domain is linked to ZO-1, a cytosolic scaffold protein, which anchors the TJ to the perijunctional actomyosin ring and thereby builds a connection to the actin cytoskeleton, which is critical to the preservation of TJ structure and function [2,3]. The extracellular loops interact homophilically or heterophilically with claudins of neighboring cells, resulting in organ- and tissue-specific paracellular barrier properties [4,5,6].

An adequate function of TJ protein expression and interaction is crucial for preventing the passage of antigens and pathogens from chyme into the blood circulation [7]. Altered claudin expression leads to TJ dysfunction, resulting in increased paracellular permeability and decreased barrier properties, which are associated with several pathological alterations from gastritis to gastric cancer [7,8]. TJ proteins are described not as static but as highly dynamic structures, undergoing constant remodelling processes triggered by both physiological and pathophysiological stimuli [9]. Damage to the stomach mucosa is often caused by food ingredients like allergens and nitrate, as well as contaminants like chemicals or medications such as non-steroidal anti-inflammatory drugs (NSAIDs) [8,10]. A side effect of NSAIDs is the suppression of the stomach mucosa protection by reducing the synthesis of prostaglandin [11]. Natural supplements, including traditional Chinese medicine formulas and other plant-based compounds, are described as protective for the gastric mucosa [12,13]; however, information about the effects of these plant compounds on epithelial integrity remains scarce.

In previous studies, we verified direct effects of secondary plant compounds on stomach-specific claudins-4, -5, and -18.2 expressed in *Xenopus laevis* oocytes [14,15]. Cinnamic acid and caffeic acid are polyphenols, a group of secondary metabolites in plants, with their primary source being plant-based foods like fruits, vegetables, leaves, flowers, and beverages like tea or coffee. They consist of an aromatic ring and one or more hydroxy groups, thus containing phenolic and acrylic functional groups [16,17]. In plants, they serve various functions, from protecting against pathogens and microbes to attracting pollinators. Due to this wide range of biological activities, they are of particular interest, especially regarding their antimicrobial, antioxidant, anti-inflammatory, and antineoplastic properties for potential therapeutic purposes [18,19].

The aim of this study was to investigate the impact of cinnamic acid and caffeic acid on native stomach mucosa in a more complex ex vivo model with a focus on epithelial tightness and alterations of claudin expression. To this end, Ussing chamber experiments were performed to measure transepithelial electrical resistance in porcine stomach mucosa incubated with cinnamic acid or caffeic acid, followed by immunoblotting and confocal laser scanning microscopy of tight junction proteins. To observe potential effects of cinnamic acid and caffeic acid not only on the physiological mucosa but also on challenged stomach epithelium, an indomethacin-induced gastric epithelium barrier impairment model for the Ussing chamber was developed.

## 2. Materials and Methods

### 2.1. Tissue Preparation

Samples from the stomach mucosa were taken from pig carcasses from a conventional slaughterhouse in Germany (Lehr- und Versuchsanstalt für Tierzucht und Tierhaltung, Ruhlsdorf, Germany, LaGeSo No. 2026-020-StN). Directly after slaughter, the stomach was withdrawn, carefully opened, and chyme was removed. After a short survey of the mucosa regarding macroscopic alterations, healthy corpus tissue was collected. Therefore, the mucosa was stripped carefully from the Tunica muscularis gaster, and remaining connective tissue was removed from the mucosa. Subsequently, the tissue was kept in ice-cold buffer solution #1 for transport. Before transportation, the buffer was adjusted to pH 7.4 and gassed with 95% oxygen and 5% carbon dioxide.

### 2.2. Buffer Solutions

All chemicals were purchased from Carl Roth GmbH, Karlsruhe, Germany.

### 2.3. Ussing Chamber Experiments

The stomach mucosal tissue was cut into smaller pieces and carefully mounted into the chambers filled with buffer solution #1 (Table 1). Before mounting of the tissue, for correction of solution resistance and self-potential of the electrodes, the PD (transepithelial potential difference) was measured in a complete experimental setup for each chamber and was incorporated into TEER calculations automatically. After an equilibration period of 45 min, the buffer was exchanged on the serosal side with the same buffer solution, and samples were gassed with 95% oxygen and 5% carbon dioxide at 37 °C. For the mucosal side, buffer solution #2 (Table 1), adjusted to pH 4.0, was added, and gassing on the mucosal side was performed with 100% oxygen at 37 °C. Buffer volume at each side was 10 mL and the buffer exposed area of the tissue was 0.96 cm^2^.

Following this, indomethacin, cinnamic acid, and caffeic acid (Sigma Aldrich, Taufkirchen, Germany), dissolved in DMSO, were added, resulting in a DMSO concentration of 0.1% for every condition. Respectively, 0.1% DMSO was added to the controls.

As depicted in Table 2, substances were either applied to the serosal or mucosal side, and transepithelial electrical resistance R_t_ [Ω · cm^2^] was constantly recorded during the whole experimental duration of 5 h. Electrophysiological parameters of the tissue were controlled via voltage clamp (Biomedical Instruments, Munich, Germany).

After 3 h, a buffer exchange was performed to ensure the vitality of the tissue. At the end of the incubation period of 5 h, vitality testing was performed by adding barium chloride to both sides at concentrations of 500 mmol/L. An increased TER after 15 min to at least 25% was defined as the cut off for vitality. During the experiment, the pH of the buffer solutions was controlled hourly.

For comparative analysis, the values of TEER were calculated relative to the resistance before adding the different compounds. This initial resistance was set to 100%. Tissue samples with a decreased resistance to less than 60% after 5 h, with an initial resistance under 20 Ω · cm^2^ or with an inadequate reaction to vitality testing, were excluded from further analysis.

Following the Ussing chamber experiments, tissue samples were removed from the Ussing chamber and cut into two halves: The first half was cryopreserved in liquid nitrogen for protein extraction and stored at −80 °C until further processing. The second half was fixed in 4% paraformaldehyde at room temperature for 4 h, followed by washing in PBS with Ca^2+^ and Mg^2+^ (PBS +/+) and transferred into PBS +/+ containing 25 mM glycine and 0.1% NaN_3_. Tissue samples were then stored at 4 °C until further processing for immunofluorescence staining, as described below.

### 2.4. Membrane Protein Extraction and Quantification

For membrane protein extraction, cryopreserved tissue samples were homogenized in RIPA buffer (25 mM HEPES pH 7.6, 25 mM NaF, 2 mM EDTA, 1% sodium dodecyl sulfate (10%), H_2_O, and enzymatic protease inhibitors (Complete EDTA-free, Boehringer, Mannheim, Germany)) by repeated crushing with 2 steel balls followed by centrifugation for 1 min at 16,000× *g*. The supernatant was centrifugated again for 15 min at 15,000× *g* at 4 °C (sigma 3–30 ks, Sigma-Aldrich, Munich, Germany). Then, the supernatant was used for protein quantification by utilizing the Bio-Rad DC Protein Assay (Bio-Rad Laboratories GmbH, Munich, Germany). The protein extracts were stored at −80 °C.

### 2.5. Immunoblots

Protein extract samples were prepared for immunoblotting by mixing 20 µg protein with Laemmli buffer (Bio-Rad Laboratories GmbH, Munich, Germany) and β-mercaptoethanol (AppliChem, Darmstadt, Germany), followed by denaturation at 95 °C for 5 min. For detection of claudin-2 and claudin-18, 9 mol/L urea (Carl Roth GmbH, Karlsruhe, Germany) was added additionally, and denaturation was performed at 55 °C for 6 min.

Following this, the protein lysates were loaded onto 10% TGX Stain-Free FastCast gels (Bio-Rad Laboratories GmbH, Munich, Germany) for electrophoresis (60 min at 150 V), followed by electroblotting (90 min at 100 V) for protein transfer onto PVDF membranes (Bio-Rad Laboratories GmbH, Munich, Germany). Subsequently, the membranes were blocked in blocking solution containing 5% milk in Tris-buffered saline with 0.1% Tween 20 for 60 min. Claudin-1, -2, -3, -4, -5, and -18 were detected with specific primary antibodies at concentrations according to the manufacturer’s recommendation (Invitrogen, Life Technologies, Carlsbad, CA, USA) at 4 °C overnight (Table 3). The next day, the membranes were incubated with peroxidase-conjugated goat anti-rabbit and anti-mouse antibodies (#7074, #7076, Cell Signaling Technology, Danvers, MA, USA) at room temperature for 45 min. Afterwards, detection solution (Clarity Western ECL Blotting Substrate, #1705061, Bio-Rad Laboratories GmbH, Munich, Germany) was added to the membranes for detecting the antibodies using a ChemiDoc MP Luminescence imager (ChemiDoc MP, Munich, Germany).

For densitometric analysis, the immunoblotting bands were normalized based on the total protein amount and analysed by comparison to the control group employing the manufacturer’s analysis software (ImageLab version 5.1, BioRad, Hercules, CA, USA), as reported recently [20,21].

### 2.6. Immunofluorescence Staining

The samples were transferred into increasing alcohol concentrations for dehydration (70%, 80%, 90%, 96%, 100%), followed by xylol, paraffin, and subsequent embedding in paraffin blocks. Sections of 5 µm thickness were prepared (Leica RM 2245 microtome, Leica Microsystems, Heidelberg, Germany), transferred to a water bath at 38 °C, and then placed on slides until further processing.

For deparaffinization, the slides were placed in xylol followed by washing steps in decreasing alcohol concentrations for rehydration. Afterwards, the slides were heated in 10 mM citrate buffer (Tri-sodium citrate (dihydrate) 2.94 g in 1 L distilled water, pH adjusted to 6.0 with 1.0 N HCl) at approximately 95 °C for 45 min, followed by permeabilization in 0.5% Triton X-100 in PBS +/+. To create a hydrophobic barrier, the mucosa sections were outlined with a PAP pen (Kisker Biotech GmbH & Co. KG, Steinfurt, Germany). The sections were then blocked in PBS containing 5% goat serum and 1% bovine serum albumin at room temperature for 30 min. Subsequently, the samples were incubated with primary antibodies raised against claudin-2, -3, -5, -18, ZO-1, and occludin (Invitrogen, Life Technologies, Carlsbad, CA, USA) at 4 °C overnight (Table 3). The following day, the slides were washed multiple times in blocking solution before incubation with secondary antibodies (goat anti-rabbit Alexa Fluor-488 #7074, goat anti-mouse Alexa Fluor-594 #7076, 1:1000, Cell Signaling Technology, Danvers, MA, USA) at 37 °C for 1 h. Afterwards, the slides were mounted with ProTaqs Mount Fluor with DAPI (Biocyc, Luckenwalde, Germany) and examined using a Zeiss 710 confocal laser scanning microscope (Zeiss, Oberkochen, Germany).

### 2.7. Statistical Analysis

Statistical analysis was performed with SPSS version 32. Data are presented as box blots representing the first quantile (25%), the median (50%), and the second quantile (75%). The whiskers show the 10th and 90th percentile. *n* is the number of animals used for the experiments. The Shapiro–Wilk test was used for checking normal contribution, followed by Kruskal–Wallis test and Wilcoxon test for non-parametric data to compare the experimental groups with the control group. Values of *p* < 0.05 are determined as statistically significant (presented as * *p* < 0.05, ** *p* < 0.01, *** *p* < 0.001).

## 3. Results

### 3.1. Impact of Indomethacin, Cinnamic Acid and Caffeic Acid on Stomach Mucosa Barrier Properties

Transepithelial electrical resistance (TER) was measured continuously for 5 h after exposure to 400 µmol/L indomethacin, 500 µmol/L cinnamic acid, 500 µmol/L caffeic acid, or combinations of indomethacin with cinnamic acid or caffeic acid. All experiments were conducted at a pH of 4.0 on the mucosal side and 7.4 on the serosal side. To exclude pH-dependent effects on the epithelial barrier function, a control group with a pH of 7.4 on the mucosal and serosal side was tested in addition. For each condition, the TER at 0 h was set to 100%. Results are presented as mean ± standard error.

After 5 h, the TER of the control group at pH 7.4 decreased to 90.83 ± 4.23% (*n* = 14), while the TER of the control group at pH 4.0 decreased to 81.21 ± 3.69% (*n* = 24). However, no significant pH-dependent effect was observed during the experimental period. Incubation with 400 µmol/L indomethacin for 5 h reduced the TER to 67.36 ± 3.69% (*n* = 27), which was significantly lower compared to the ctrl pH 4.0 (*p* = 0.0001). In contrast, treatment with 500 µmol/L cinnamic acid (78.49 ± 5.71% after 5 h, *n* = 15) or caffeic acid (81.47 ± 4.92% after 5 h, *n* = 13) alone showed no significant effects (*p* = 0.46; *p* = 0.65) compared to the control at pH 4.0.

A combination of 400 µmol/L indomethacin with 500 µmol/L cinnamic acid resulted in a decreased TER of 65.06 ± 4.5% (*n* = 11) after 5 h. Compared to incubation with indomethacin alone, the addition of cinnamic acid showed no significant effect. In contrast, treatment with a combination of 400 µmol/L indomethacin and 500 µmol/L caffeic acid mitigated the decreasing effect of indomethacin, resulting in a TER of 81.0 ± 3.12% (n = 17, *p* = 0.002) after 5 h. Results of TER measurements in all experimental groups after 5 h are presented in Figure 1.

Over time, incubation with 400 µmol/L indomethacin led to a decrease in TER compared to respective controls already after 1 h (*p* = 0.046; ctrl: TER = 95.88 ± 2.98%, n = 24; indomethacin: TER after 1 h = 82.29 ± 3.47%, n = 27). This effect intensified during the following hours (2 h: *p* = 0.028; ctrl: TER = 87.44 ± 3.58%, n = 24; indomethacin: TER = 72.01 ± 3.59%, n = 27; 3 h: *p* = 0.11; ctrl: TER = 84.03 ± 2.75%, n = 24; indomethacin: TER = 71.58 ± 3.41%, n = 27; 4 h: *p* = 0.003; ctrl: TER = 88.73 ± 3.34%, n = 24; indomethacin: TER = 69.16 ± 2.65%, n = 27; Figure 2).

The effect of indomethacin was mitigated by the addition of 500 µmol/L caffeic acid over time (1 h: *p* = 0.36, indomethacin: TER = 82.29 ± 3.47%, n = 27; indomethacin + caffeic acid: TER = 89.63 ± 3.54%, n = 17; 2 h: *p* = 0.039, indomethacin: TER = 72.01 ± 3.59%, n = 27; indomethacin + caffeic acid: TER = 84.35 ± 3.44%, n = 17; 3 h: *p* = 0.068, indomethacin: TER = 71.58 ± 3.41%, n = 27; indomethacin + caffeic acid: TER = 79.89 ± 3.58%, n = 17; 4 h: *p* = 0.004, indomethacin: TER = 69.16 ± 2.65%, n = 27; indomethacin + caffeic acid: TER = 81.71 ± 3.92%, n = 17; Figure 2).

### 3.2. Immunoblotting of TJ Proteins

After incubation in the Ussing chamber, tissue samples were collected for immunoblotting to observe changes in TJ protein expression under conditions with altered TER. Controls were set to 100%, and tissue samples treated with indomethacin, caffeic acid, or a combination of both were compared to the controls. Western blotting signals and densitometry results are shown in Figure 3. Original Western Blot images for Figure 3A are found in Supplementary Materials. There were no significant changes in claudin-1 (n = 4) and claudin-4 (n = 3–7) expression, but claudin-2, -3, -5, and -18 expression was affected by incubation with indomethacin. Expression of claudin-2 and claudin-18 decreased to 31.94 ± 11.26% (*p* = 0.0038, n = 5) and 46.44 ± 9.47% (*p* = 0.043, n = 5), while claudin-5 expression increased to 198.04 ± 33.07% (*p* = 0.0313, n = 6). Additional treatment with caffeic acid mitigated the effects of indomethacin, resulting in expression levels of 123.97 ± 38.03% (n = 4) for claudin-2, 78.71 ± 12.79% (n = 5) for claudin-5, and 78.3 ± 24.35% (n = 5) for claudin-18 compared to controls. Treatment with indomethacin as well as the combination of indomethacin and caffeic acid decreased claudin-3 expression to 46.6 ± 3.52% (n = 4, *p* = 0.0006) and 38.26 ± 13.77% (n = 5, *p* = 0.011), respectively. Incubation with caffeic acid alone had no significant effects on TJ protein expression.

### 3.3. Confocal Laser-Scanning Immunofluorescence Microscopy

To analyze the localisation of TJ proteins that had shown expression alterations in immunoblotting, tissue samples were stained with antibodies raised against specific TJ proteins for immunohistochemistry. Additionally, the TJ proteins occludin and ZO-1 were stained to observe changes in their expression and co-localisations with other TJ proteins. In accordance with the immunoblot results, an altered expression pattern for claudin-2 was observed after incubation with indomethacin. There was a delocalisation of claudin-2 from the membrane to the cytosol, resulting in no clear signal within the cell membrane. In controls, claudin-18 is particularly expressed in the lateral membrane, and after incubation with indomethacin, this expression pattern changed to an enhanced localization in every part of the membrane (Figure 4A). Tissue samples challenged with indomethacin showed a clear delocalization of claudin-3 from the membrane to the nucleus. While treatment with the NSAID alone resulted in a loss of claudin-3 within the membrane, additional incubation with caffeic acid led to a remaining signal for this protein within the membrane. Localization of occludin was diffuse within the cytosol under all conditions, but after treatment with indomethacin, there was an enhanced localization in the apical parts of the cells (Figure 4B). Strong signals for claudin-5 were observed in tissues treated with indomethacin, which is in accordance with the immunoblot results. In controls, ZO-1 was localized especially within the apical membrane, and in challenged tissue samples, it was delocalized to the cytosol (Figure 4C).

### 3.4. Vitality Testing for Porcine Stomach Mucosa

For verification of tissue sample vitality, barium chloride was added to both sides of the tissue at the end of the Ussing chamber experiments. This led to an increased TER after 15 min. As no significant differences in barium chloride response were observed, unaltered vitality between the different groups was ensured (Figure 5).

## 4. Discussion

The gastric epithelium is described as a tight epithelium, and its integrity is crucial for preventing the diffusion of protons, ions, digestive enzymes, food substances, toxins, pathogens, heavy metals, pharmaceuticals, and other substances from the luminal chyme into the bloodstream [22]. Several pathogens and pharmaceuticals have the ability to damage the stomach epithelium by manipulating the regulatory functions of TJs, thereby disrupting their structure. This leads to barrier dysfunction and contributes to pathological processes. For example, infections with *H. pylori* result in increased paracellular permeability by activating myosin light chain kinase (MLCK), which phosphorylates MLC, leading to contraction of perijunctional actin and consequently increasing paracellular permeability through distension of transmembrane TJ strands [4,23]. Non-steroidal anti-inflammatory drugs (NSAIDs), such as indomethacin, reduce inflammation and pain, but at the same time, they suppress the secretion of bicarbonate and gastric mucus, thereby decreasing stomach protection against acidic pH by reducing prostaglandin synthesis [10].

At the same time, various substances may provide strengthening effects on the stomach mucosa. In particular, a plant-based diet provides a high daily intake of several secondary plant compounds, such as polyphenols. One subgroup, namely phenolic acids, is known to act on various cellular pathways to enhance the integrity of tight junctions and alleviate intestinal barrier disruption. Specifically, chlorogenic acid, an ester of caffeic acid and ferulic acid, improves intestinal integrity and increases protein levels for occludin and ZO-1 in the jejunum and colon of weaned rats challenged with lipopolysaccharide [24]. Moreover, it increased TER in Caco-2 and LS174T cells by inhibition of MLCK and Rho-associated kinase 1 (ROCK1) activation [25]. Extract of *Petasites japonicus* leaves, containing, among other compounds, chlorogenic acid and caffeic acid, alleviated dextran sulfate sodium-induced colitis in mice by upregulation of ZO-1, occludin, and claudin-1 protein expression [26]. Dietary supplementation of cinnamic acid increased mRNA expression levels of these TJ proteins in the colonic epithelium of weaning piglets [27]. Other polyphenols from the flavonoid subgroup were also described as participating in barrier regulation. For example, quercetin increases TER in combination with transcriptional regulation of claudin-4 and the assembly of ZO-2, occludin, and claudin-1 by direct inhibition of protein kinase C delta in Caco-2 cells [28].

While numerous studies have investigated the effects of secondary plant compounds on the intestinal barrier, insights into their effects on the gastric epithelium remain insufficient. In a previous study, the strengthening effects of two phenolic acids, cinnamic acid and caffeic acid, on gastric TJ proteins claudin-4, -5, and -18 expressed in *Xenopus laevis* oocytes were identified [14]. In the present study, the effects of these two secondary plant compounds were observed on native and indomethacin-challenged porcine stomach mucosa with regard to epithelial tightness and tight junction protein expression. Measurement of tightness refers to the measurement of transepithelial electrical resistance, and an established technique for measuring the TER in native porcine tissue ex vivo is the Ussing chamber [21,29,30]. This method enables the development of new models for examining not only native tissue but also challenged tissue [31]. One limitation of ex vivo Ussing chamber experiments is the incubation time due to tissue viability.

The NSAID indomethacin is a widely used model to induce gastric ulcers in rats [12,32]. High concentrations of indomethacin have been described as a potential means to induce gastric ulcers within a few hours [13], making it a suitable agent for damaging the stomach epithelium in a time-limited model. In Ussing chamber experiments with porcine stomach mucosa, the incubation with 400 µmol/L indomethacin led to a significantly decreased TER already after 1 h, and this effect intensified during the total experimental time of 6 h. After 2 h, additional treatment with 500 µmol/L caffeic acid began to significantly mitigate this decline of TER. Treatment with a combination of indomethacin and cinnamic acid had no such effect. Cinnamic acid features only one hydroxy group, whereas caffeic acid, a hydroxycinnamic acid derivative, has two additional hydroxy groups, so caffeic acid is more reactive than cinnamic acid [33]. This difference may explain the results of TER measurements in our study. Daily average intake of caffeic acid in humans depends on the individual diet, including fruits and vegetables and the consumption of coffee and herbal teas, and ranges from 25 to 500 mg caffeic acid per day [34]. Therefore, information about the concentration of caffeic acid reached inside the stomach is scarce. Inside the colon, caffeic acid concentrations from 50 µmol/L up to 126 µmol/L are reached [35]. Calculations of the concentration of caffeic acid in 500 mL of brewed coffee result in a concentration of about 293 µmol/L (concentration of caffeic acid in coffee = 87 mg/100 g [34], use of 30 g coffee for brewing 500 mL, molar mass of caffeic acid = 180.16 g/mol). Thus, the tested concentration of 500 µmol/L cinnamic acid or caffeic acid represents a superior high intake in a dietary context, or rather an intake of potentially pharmaceutical amounts. The effect of caffeic acid and cinnamic acid in a dose-dependent manner should be included in further investigations regarding the therapeutic potential of these plant-based compounds.

Immunoblotting and confocal laser scanning immunofluorescence provide deeper insights into the effects of indomethacin and caffeic acid on gastric epithelium TJ proteins.

Claudin-1 is known as a member of the claudin family with sealing properties [29]. The expression of claudin-1 within the porcine stomach mucosa was verified by immunoblotting; exposure to indomethacin, caffeic acid, or a combination showed no significant differences in claudin-1 expression.

Another claudin, known as a barrier-forming protein in, e.g., kidney, gallbladder, and intestinal cells, which is also expressed in porcine stomach epithelium, is claudin-3 [36]. In this study, incubation with indomethacin and an indomethacin–caffeic acid combination led to decreased expression. Confocal laser scanning immunofluorescence revealed that incubation with indomethacin alone or in combination with caffeic acid led to an altered localization of claudin-3 within the cell. There was a decreased expression of claudin-3 in the cell membranes, but a high expression within the nucleus, which was not observed in the controls or in the tissue incubated with caffeic acid alone. A mislocalization of claudin-3 to the nucleus was observed in colon cancer before [37], which indicates that the protein expression pattern of claudin-3 changes under pathological conditions. Therefore, translocation to the nucleus leads to a loss of its sealing function within the cell membrane.

Claudin-4, characterized as a barrier-forming claudin [38], showed weak expression in porcine stomach mucosa by immunoblotting, and there were no significant effects of incubation with indomethacin or caffeic acid. However, expression of claudin-4 was increased in challenged stomach epithelia compared to the controls. The role of claudin-4 in general is inconsistent, as overexpression of claudin-4 in MDCK II cells was reported to result in an increased TER through a selective decrease in paracellular sodium permeability, whereas in LLC-PK 1 cells, no effect on TER was observed [39]. Claudin-4 showed sealing properties in stomach mucosa, as claudin-4-overexpressing gastric cancer cells exhibited increased barrier function of the gastric TJs [40], and *H. pylori* infection in human gastric HE-20 monolayers led to decreased claudin-4 expression and a redistribution from the membrane to the cytosol [41].

In this study, claudin-5 expression was increased after incubation with indomethacin and could be mitigated by adding caffeic acid. Claudin-5 is a sealing claudin, and overexpression in Caco-2 cells has been reported to cause an increased TER [42]. In stomach adenocarcinoma, claudin-5 expression is lower compared to adjacent normal tissue, but it is highly expressed in para-cancer tissue and decreases with advancing pathological stage [43]. Consequently, higher expression of claudin-5 could be an early marker for pathological processes within the epithelium induced by indomethacin, whereas caffeic acid prevents this effect.

Claudin-18 expression was also affected by incubation with indomethacin. Immunoblotting revealed a decreased expression in stomach epithelial membranes compared to controls, and confocal laser scanning microscopy showed an altered expression pattern. In controls, claudin-18 was especially expressed in epithelial membranes, but after incubation with indomethacin, the expression in the membrane decreased, indicating redistribution to the cytosol. This effect was not observed after treatment with caffeic acid and indomethacin together. In the stomach epithelium, claudin-18 participates in forming a paracellular barrier against hydrochloric acid and functions as an important proton barrier, and loss of claudin-18 is associated with proton leakage and atrophic gastritis in mice [44]. In human early gastric cancer lesions, claudin-18 was shown to be downregulated compared to the surrounding normal gastric mucosa [45]. Therefore, decreased expression after treatment with indomethacin could be a sign of early pathological events within the stomach epithelium, and caffeic acid appears to mitigate this process.

In this study, the expression of a pore-forming claudin, namely claudin-2, in porcine stomach mucosa is also verified by immunoblotting and confocal laser scanning immunofluorescence. Incubation with indomethacin led to a decreased expression, whereas caffeic acid or a combination with indomethacin had no significant effect compared to the controls. Claudin-2 is known to form paracellular channels, allowing the paracellular passage of cations that are smaller than 182 Da [46]. Claudin-2 expression in human gastric tissue was found in 25% of gastric carcinoma tissue samples and in 67.8% of adjacent tissues, while the expression in cancer tissues was significantly lower than in adjacent tissues [47]. Another study revealed no claudin-2 protein expression in chronic superficial gastritis, chronic atrophic gastritis, or intestine-type metaplasia but increased expression in the dysplasia and gastric intestine-type adenocarcinoma [48]. Thus, there are inconsistent findings regarding its physiological and pathological expression patterns in the gastric epithelium. The decreased expression after treatment with indomethacin in this study might be based on the anti-inflammatory effect of this NSAID but could also be in accordance with the findings from Song et al. [48], where there was no expression in gastritis tissue samples. Further studies are required to identify the role and function of claudin-2 within the stomach epithelium.

However, the changes in claudin expression alone cannot explain the decreasing TER in this study. As shown in immunohistochemistry results there is also an altered localization of the TJ protein ZO-1 in challenged tissue. The presence of ZO-1 is a critical determinant of barrier formation [3,49] and an increased paracellular permeability is accompanied by ZO-1 delocalization from the lateral TJ membrane to cytosolic pools [49,50].

Former studies revealed anti-inflammatory and, therewith, barrier-protective properties of caffeic acid and caffeic acid derivates like caffeic acid phenyl ester or chlorogenic acid. They are reported as effective plant compounds to reduce inflammatory mediators, including pro-inflammatory cytokines like tumor necrosis factor (TNF) [51,52,53]. A possible explanation of caffeic-acid-induced barrier-strengthening effects might be the inhibition of TNF affecting TJ protein expression [31,54]. Furthermore, inhibition of the transcription factor NF-kB and the inhibition of activation of MLCK and ROCK1 have been described, as well as a caffeic acid phenyl ester-mediated reduction in toll-like receptor 4 [25,55].

The present study did not focus on revealing the signaling pathways of caffeic acid and therefore does not provide conclusions regarding the underlying mechanisms of its gastric epithelium barrier strengthening effects, which might be addressed in future experiments.

The results indicate that indomethacin has a damaging effect on the stomach epithelium. The decreased TER in Ussing chamber experiments, as well as altered expression patterns of claudin-2, -3, -5, and -18 and changed ZO-1 localization, show that this NSAID is a potent agent to induce pathological changes within the stomach mucosa in Ussing chamber experiments. Hence, for the first time, we performed induced gastric epithelial barrier impairment experiments on the porcine stomach epithelium ex vivo. The decrease in TER is not caused by decreasing vitality of the tissue, as this was ensured by vitality testing by adding barium chloride at the end of the incubation time. Potassium channels are blocked by barium chloride, resulting in an increased TER [56]. As the application of caffeic acid alone had no effect on the stomach mucosa, incubation with a combination of indomethacin and caffeic acid mitigates all indomethacin-induced effects, except the decreased expression of claudin-3; thus, caffeic acid could provide therapeutic properties for the treatment and prevention of gastric injuries.

## 5. Conclusions

Caffeic acid mitigated the indomethacin-mediated barrier alterations in porcine stomach epithelial tissue, which indicates a strengthening effect of caffeic acid on indomethacin-challenged porcine gastric mucosa ex vivo. Therefore, caffeic acid could provide benefits as a plant-based drug in prevention and treatment of gastritis. Moreover, our novel Ussing chamber approach of employing indomethacin allows us to examine potential effects of different compounds on challenged stomach epithelium ex vivo as a model of gastric epithelial barrier impairment.

## Figures and Tables

**Figure 1:**
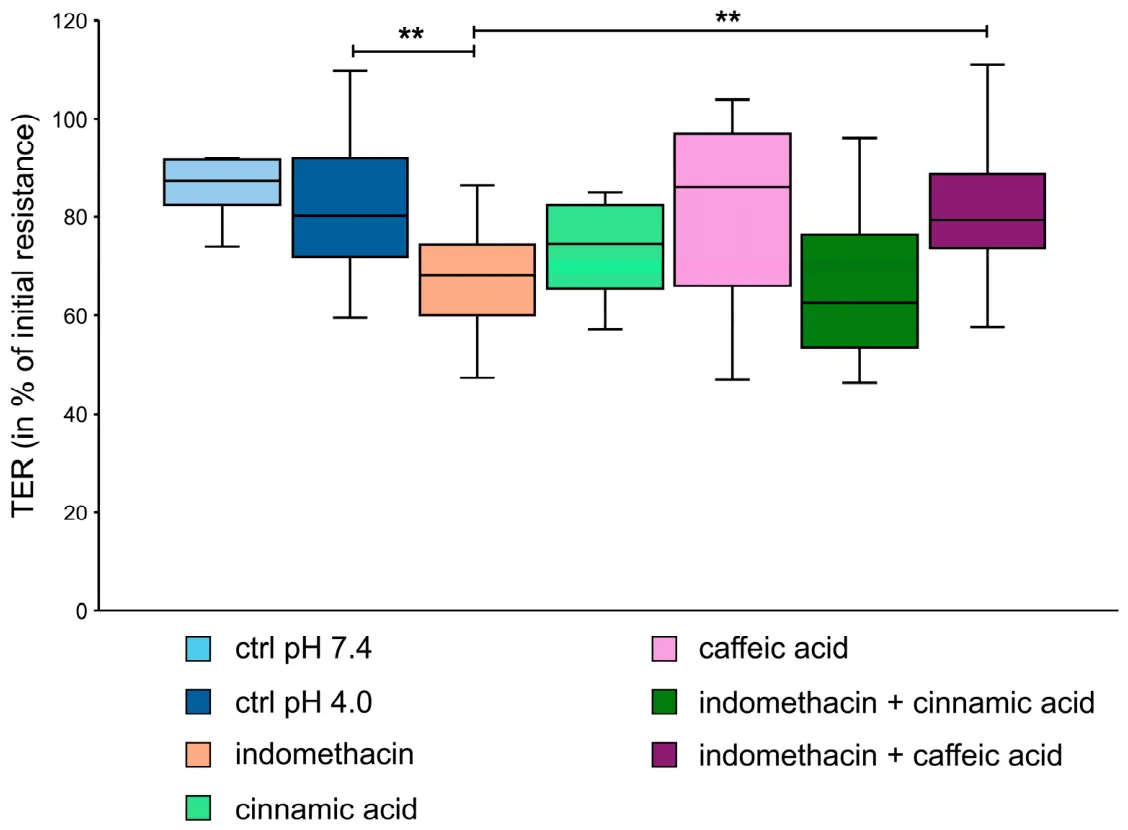
TER measurements by the Ussing chamber technique. Effects of indomethacin, cinnamic acid, caffeic acid, or combinations on transepithelial electrical resistance (TER) after 5 h. Data are presented as a percentage of initial TER (** *p* < 0.01).

**Figure 2:**
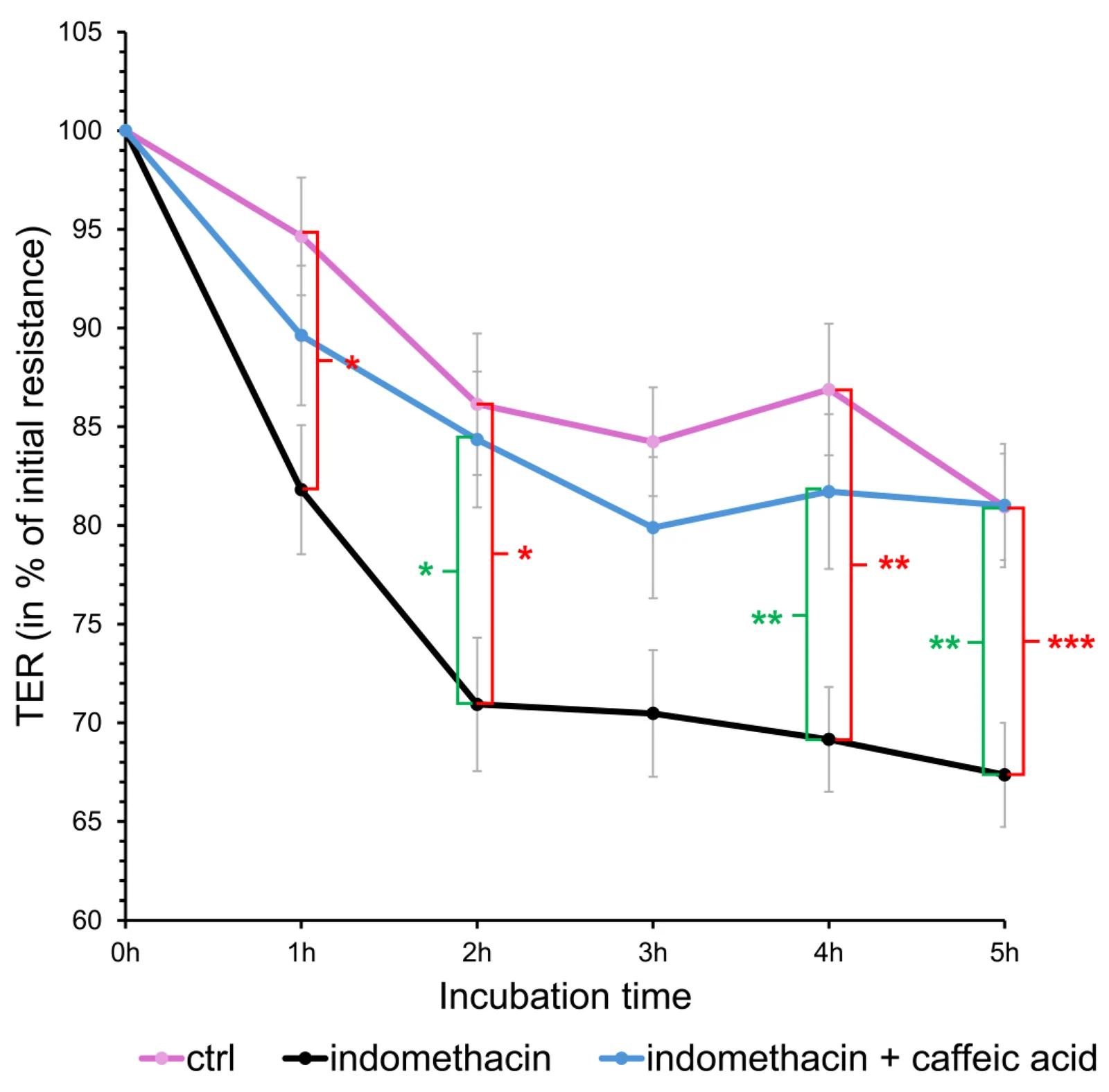
Time dependent effect of 400 µmol/L indomethacin on stomach epithelium compared to ctrl (n = 24–27; significance marked in red), and a combination of 400 µmol/L indomethacin and 500 µmol/L caffeic acid (n = 17–27; significance marked in green). Indomethacin was added to the serosal side and caffeic acid to the mucosal side. TER was recorded constantly for 5 h. Data are shown as mean as the percentage of TER ± SEM at 0 h (* *p* < 0.05, ** *p* < 0.01, *** *p* < 0.001).

**Figure 3:**
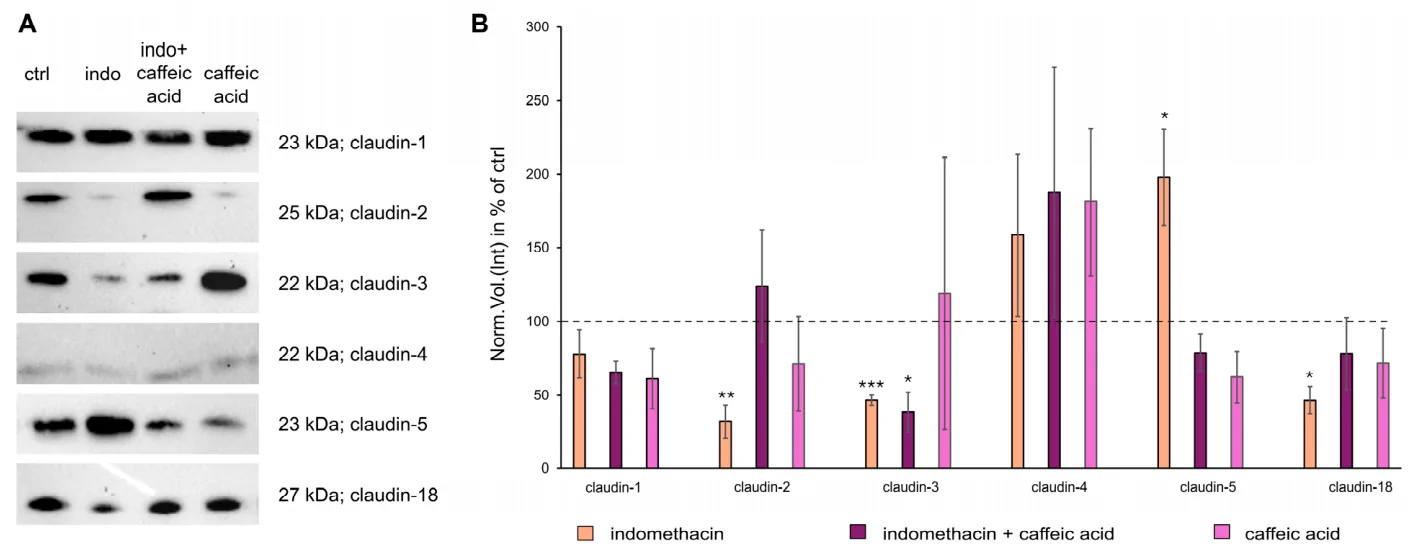
Immunoblot analysis. Representative specific signals of claudin-1, -2, -3, -4, -5, and -18 of physiological and challenged stomach epithelium at predicted sizes (**A**) and densitometry analysis compared to controls (**B**). Indomethacin led to a lower expression of claudin-2 and -18, whereas the expression of claudin-5 was increased. Incubation with indomethacin alone or in combination with caffeic acid showed a decreased expression of claudin-3 (n = 3–7, * *p* < 0.05, ** *p* < 0.01, *** *p* < 0.001). The dotted line presents the controls set to 100%. Data are presented as mean ± SEM.

**Figure 4:**
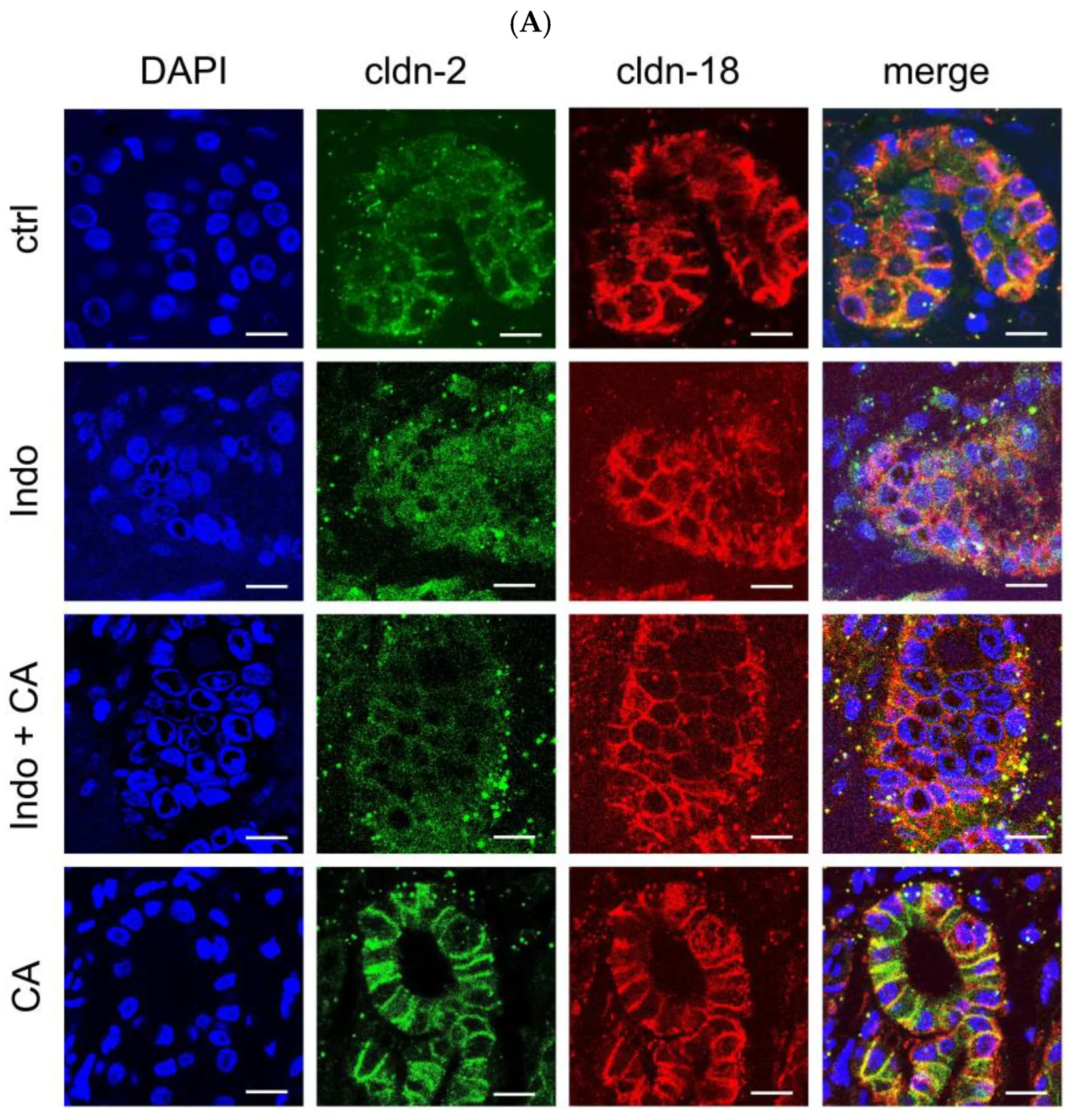

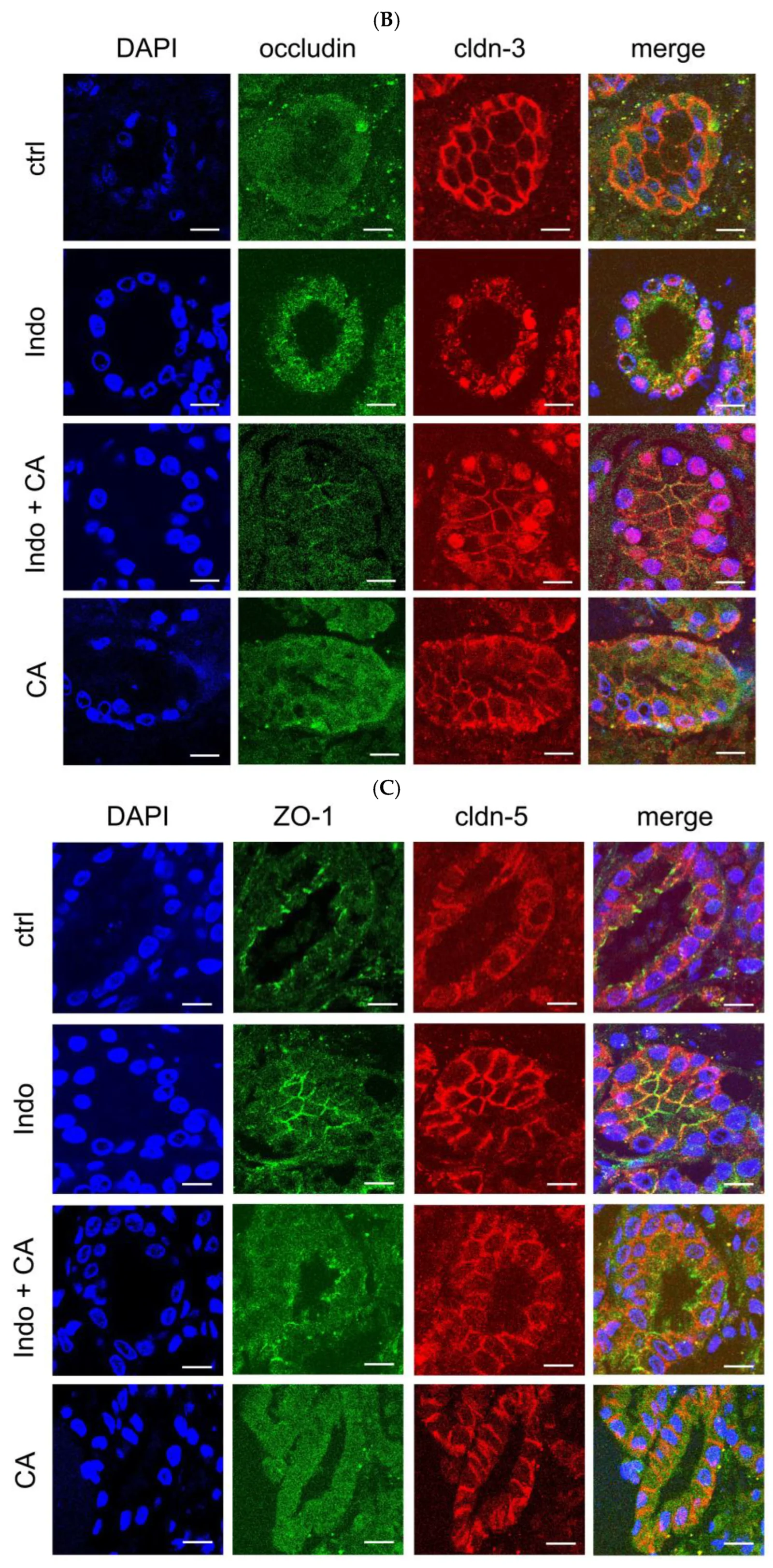
Immunofluorescent staining of affected TJ proteins within the *foveolae gastricae* of physiological and challenged stomach mucosa. Claudin-2 (green) and claudin-18 (red) (**A**), occludin (green) and claudin-3 (red) (**B**), ZO-1 (green) and claudin-5 (red) (**C**). Cell nuclei were stained blue by DAPI (scale bar = 10 µm, n = 3, representative images).

**Figure 5:**
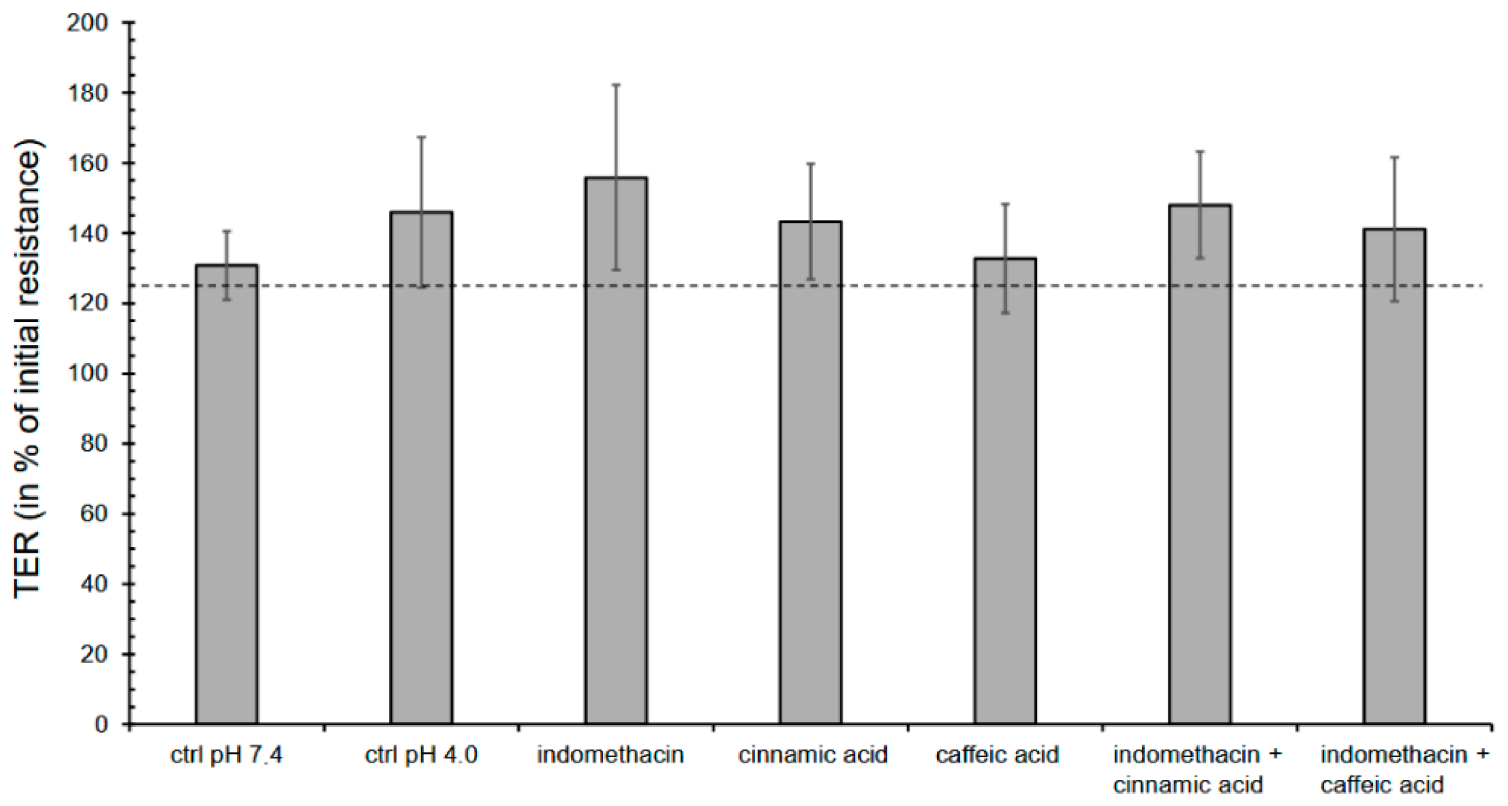
Vitality testing. TER changes 15 min after adding the vitality marker compared to the TER value 5 min before addition. The dotted line presents the cut off value at 125%; tissue with increased TER values to at least 125% 15 min after adding barium chloride was defined as vital. Data are presented as mean ± SEM.

**Table 1:** Buffer solutions No. (#) 1 and 2.

Substance	Buffer Solution #1 (Serosal) in mmol·L^−1^	Buffer Solution #2 (Mucosal) in mmol·L^−1^
Sodium chloride	119	100
Sodium bicarbonate	25	
Sodium dihydrogen phosphate	0.6	2
Di-sodium hydrogen phosphate	2.4	1
Potassium chloride	5	
HEPES	10	
Glucose	10	5
Calcium chloride	1.2	2
Magnesium chloride	1.2	1
Sodium sulphate		20
Acetic acid		40

**Table 2:** Presentation of all tested combinations.

	Mucosal		Serosal	
ctrl pH 7.4	pH 7.4		pH 7.4	
ctrl pH 4.0	pH 4.0		pH 7.4	
indomethacin 400 µM	pH 4.0		pH 7.4	+ 400 µM indomethacin
cinnamic acid 500 µM	pH 4.0	+ 500 µM cinnamic acid	pH 7.4	
caffeic acid 500 µM	pH 4.0	+ 500 µM caffeic acid	pH 7.4	
indomethacin + cinnamic acid	pH 4.0	+ 500 µM cinnamic acid	pH 7.4	+ 400 µM indomethacin
indomethacin + caffeic acid	pH 4.0	+ 500 µM caffeic acid	pH 7.4	+ 400 µM indomethacin

**Table 3:** Concentrations of specific primary antibodies.

Primary Antibody	Detected Protein	Western Blot	Immunofluorescence
Invitrogen #51-9000	claudin-1	1 µg/mL	
Invitrogen #51-6100 Invitrogen #32-5600	claudin-2	1 µg/mL	1 µg/mL
Invitrogen #34-1700	claudin-3	1:1000	1:100
Invitrogen #32-9400	claudin-4	1 µg/mL	
Invitrogen #34-1600	claudin-5	1 µg/mL	1:50
Invitrogen #700178	claudin-18	2 µg/mL	2 µg/mL
Invitrogen #33-1500	occludin		2 µg/mL
Invitrogen #33-9100	ZO-1		5 µg/mL

## Data Availability

Data are contained within the article. The datasets analyzed during the current study are available from the corresponding author upon reasonable request.

## Supplementary Materials

The following supporting information can be downloaded at: https://www.mdpi.com/article/10.3390/biom16101512/s1, Original Western Blot images for Figure 3A are found in Supplementary Materials.

## Abbreviations

The following abbreviations are used in this manuscript:

TJ	Tight junction
TER	Transepithelial resistance
ZO-1	Zonula-occludens 1
ZO-2	Zonula-occludens 2
NSAID	Non-steroidal anti-inflammatory drug
MLCK	Myosin light chain kinase
ROCK1	Rho-associated kinase 1
