# Supplementary material for: Caffeic Acid but Not Cinnamic Acid Mitigates Impairing Effects of Indomethacin in Porcine Stomach Epithelium In Vitro
Source: Biomolecules. 2026 Oct 18;16(10):1512. doi: 10.3390/biom16101512 (PMC13702404; doi:10.3390/biom16101512)
Supplement: Supplementary file 1 [file biomolecules-16-01512-s001.zip › biomolecules-4547142-supplementary.pdf]

claudin-1

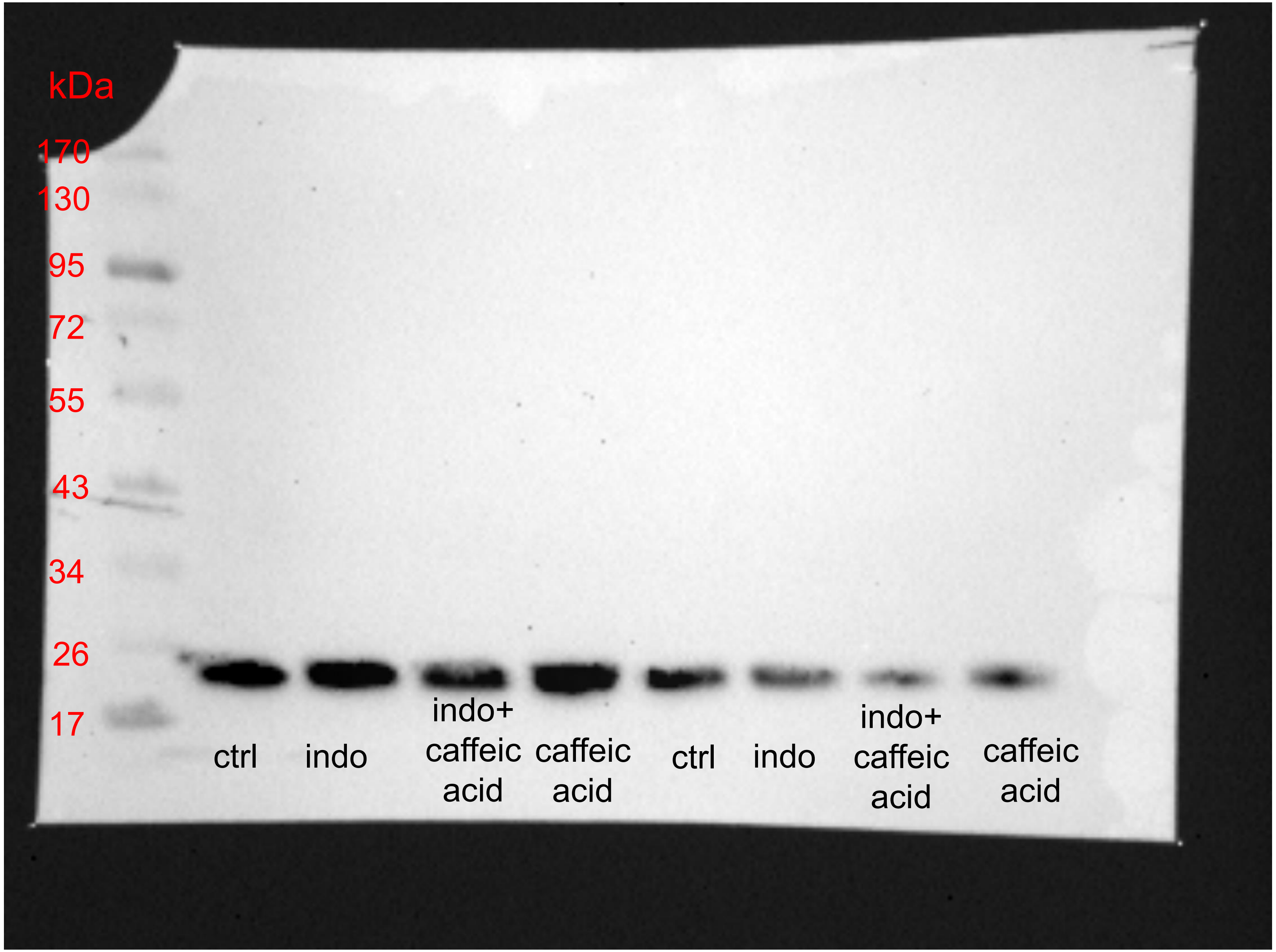
kDa
170
130
95
72
55
43
34
26
17
ctrl
indo
indo+
caffeic
acid
caffeic
acid
ctrl
indo
indo+
caffeic
acid
caffeic
acid

claudin-2

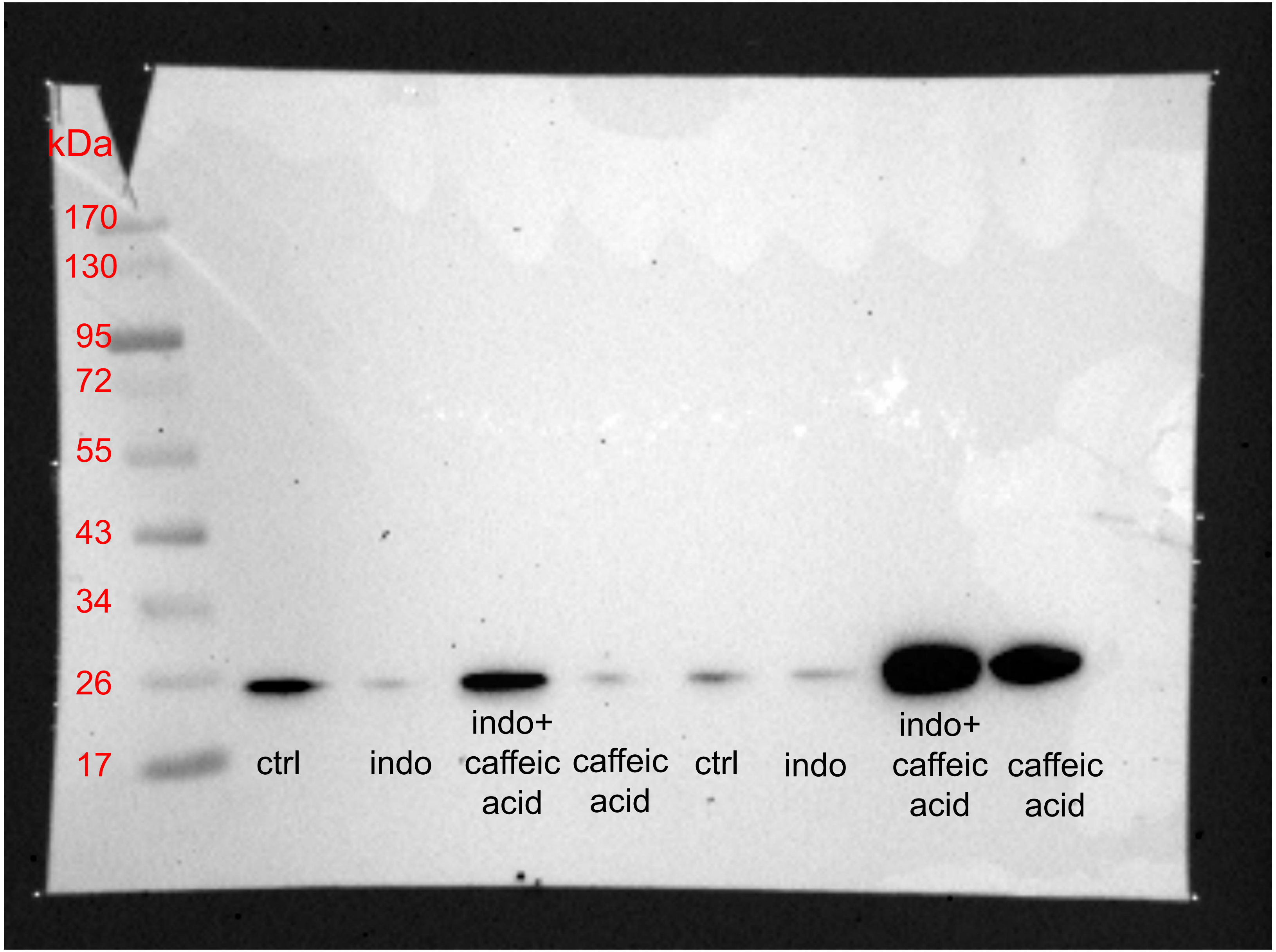

kDa
170
130
95
72
55
43
34
26
17
ctrl
indo
indo+ caffeic acid
caffeic acid
ctrl
indo
indo+ caffeic acid
caffeic acid

claudin-3

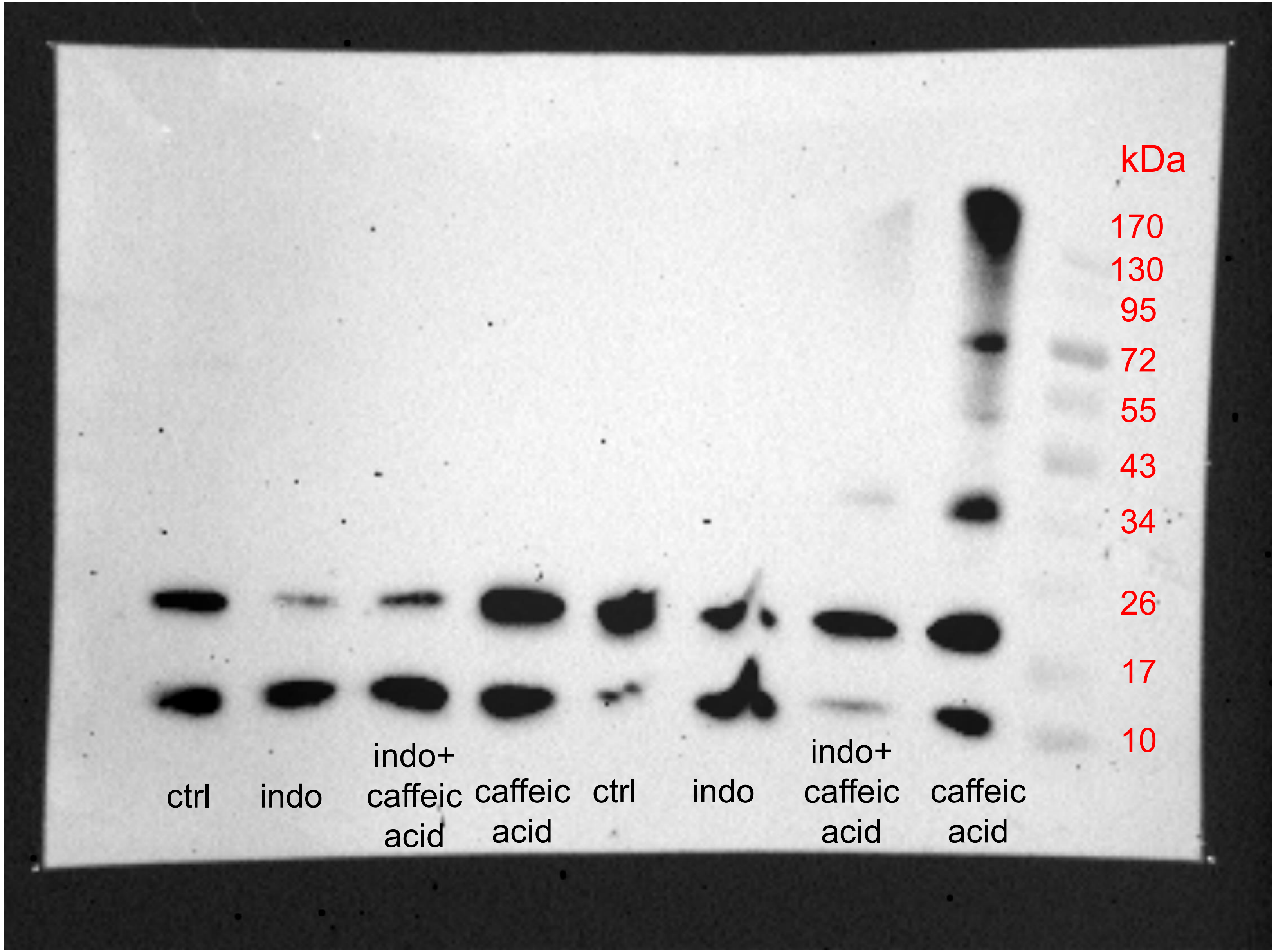

kDa
170
130
95
72
55
43
34
26
17
10
ctrl
indo
indo+
caffeic
acid
caffeic
acid
ctrl
indo
indo+
caffeic
acid
caffeic
acid

claudin-4

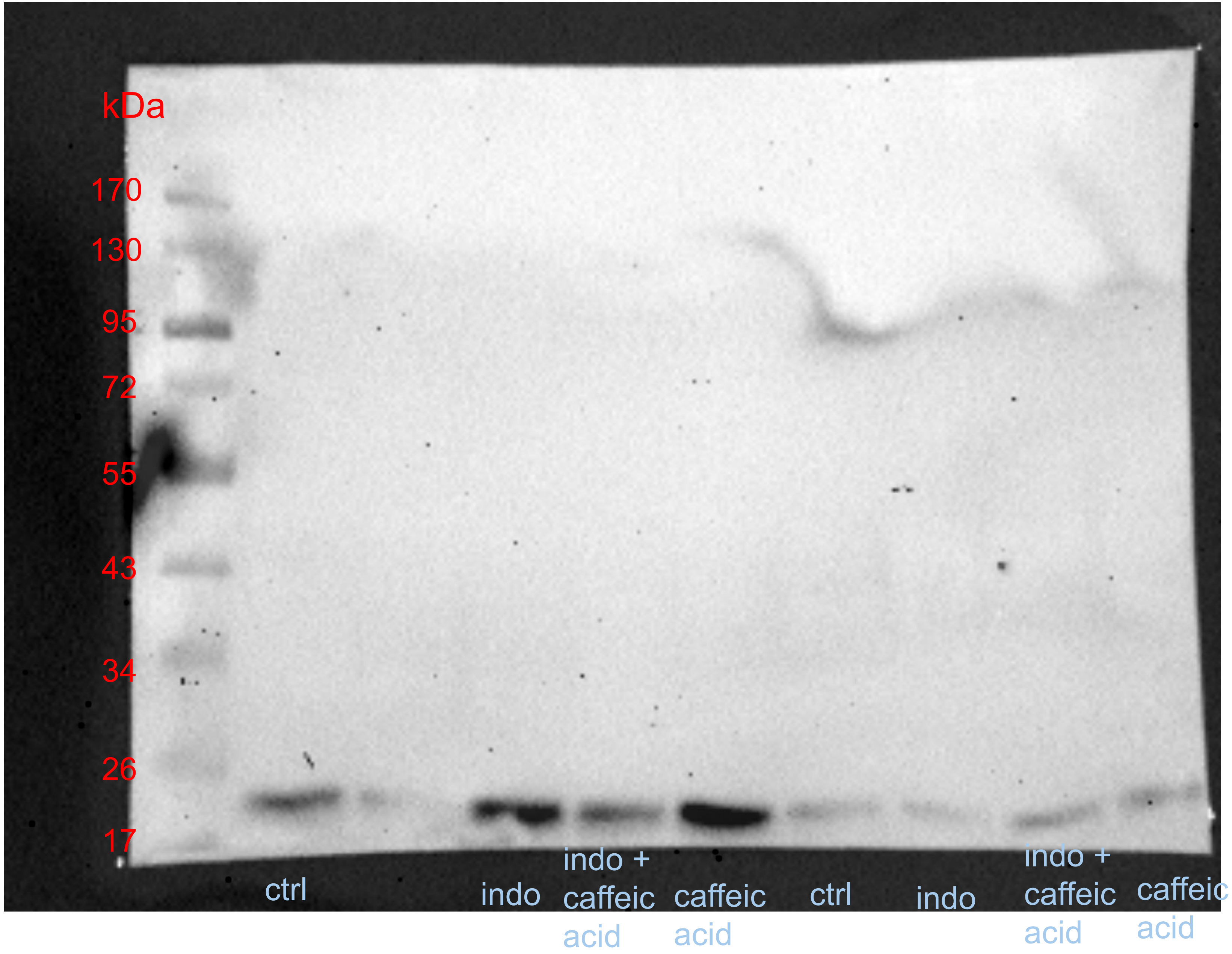
kDa
170
130
95
72
55
43
34
26
17
ctrl
indo
indo + caffeic acid
caffeic acid
ctrl
indo
indo + caffeic acid
caffeic acid

claudin-5

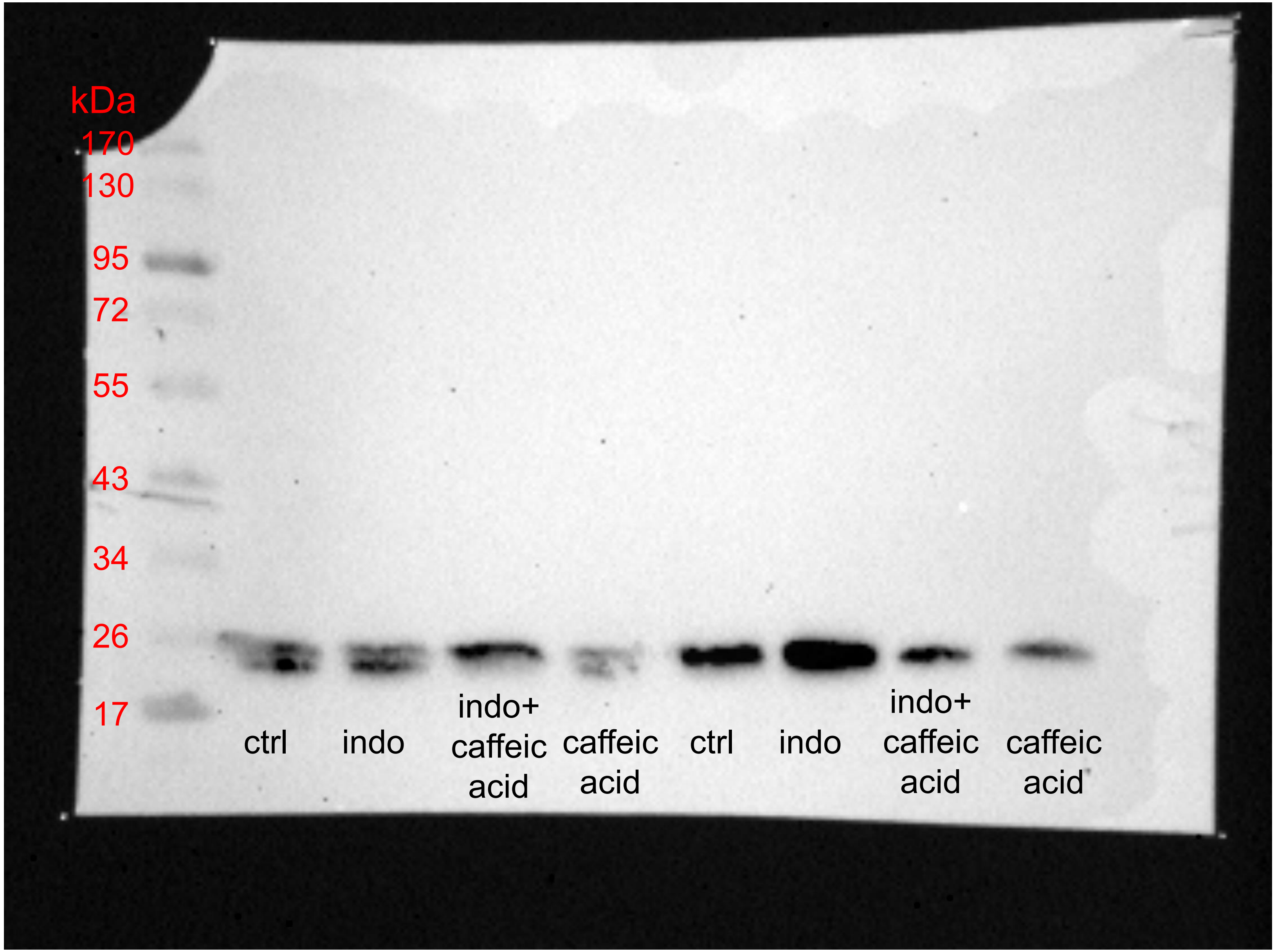
kDa
170
130
95
72
55
43
34
26
17
ctrl
indo
indo+
caffeic
acid
caffeic
acid
ctrl
indo
indo+
caffeic
acid
caffeic
acid

claudin-18

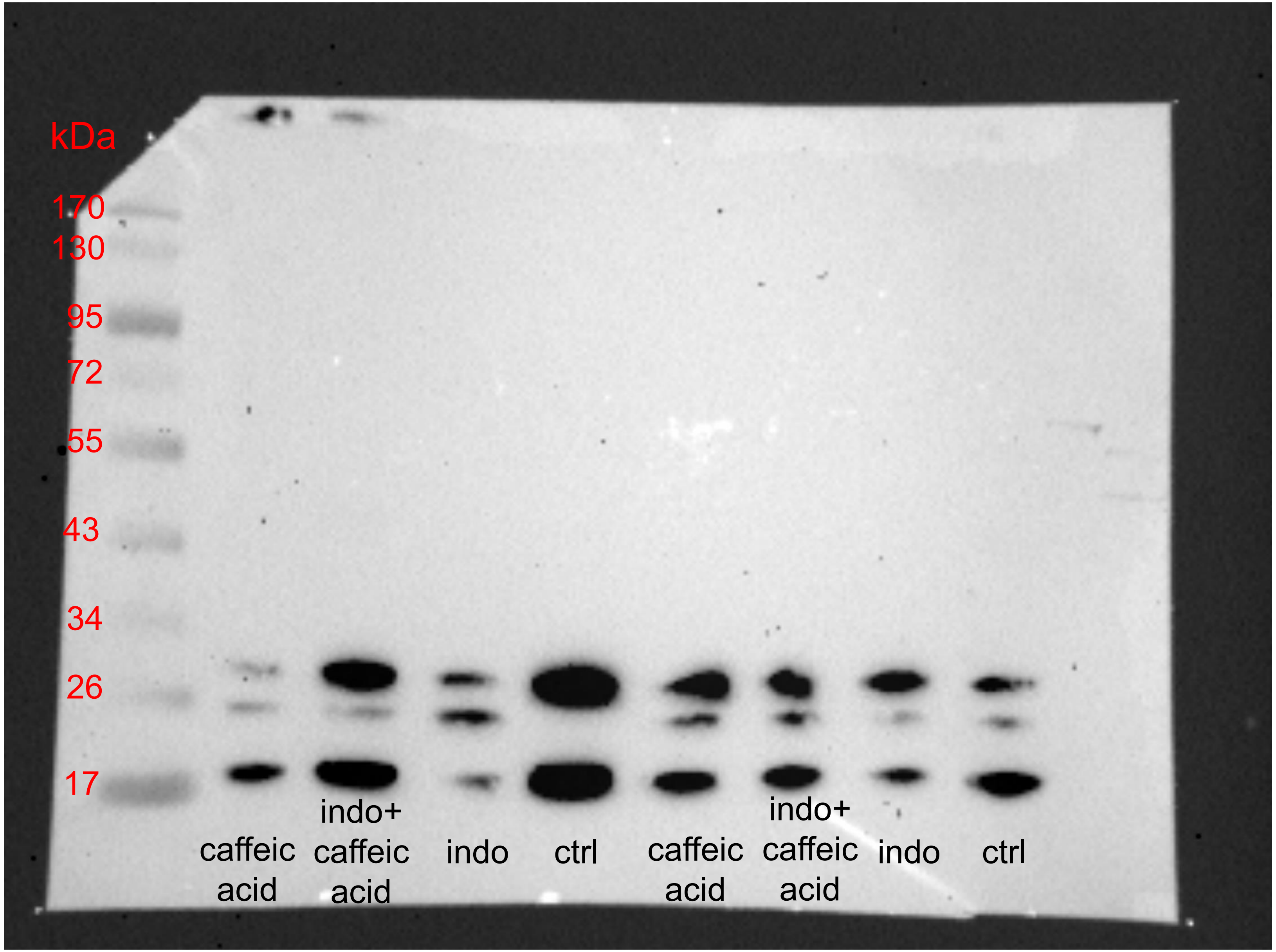
kDa
170
130
95
72
55
43
34
26
17
caffeic acid
indo+ caffeic acid
indo
ctrl
caffeic acid
indo+ caffeic acid
indo
ctrl
